# Analysis of sounds made by *Bos taurus* and *Bubalus bubalis* dams to their calves

**DOI:** 10.3389/fvets.2025.1549100

**Published:** 2025-03-18

**Authors:** Ádám Lenner, Zoltán Lajos Papp, István Komlósi

**Affiliations:** ^1^Doctoral School of Animal Science, University of Debrecen, Debrecen, Hungary; ^2^Faculty of Informatics, University of Debrecen, Debrecen, Hungary; ^3^Faculty of Agricultural and Food Sciences and Environmental Management, University of Debrecen, Debrecen, Hungary

**Keywords:** vocalization, buffalo, beef, cepstrum, mel scale bio acoustic

## Abstract

Audio and video recording techniques have advanced significantly in recent years, allowing newer opportunities for sound analysis. The grouping of cattle breeds or individuals based on the connections between their behavior and condition and their vocalizations is important from the point of view of animal welfare. Despite the numerous studies published about the acoustic characteristics of such sounds, there has not been an acoustic analysis regarding of cattle behavior and condition in isolation. The grey cattle and domestic buffalo cows, separated from their calves for a few minutes, are stressed and vocalize orally. In this study various methods were employed for the analysis of the sounds that water buffalo and grey cattle made after weaning. Differences have been found between the two species, but not between individuals. Their pitch varies over time for both species. The buffalo voice is three times more dynamic than that of the grey cattle on a logHz scale. Furthermore, a significant difference was found between relevant mel frequency cepstral coefficients adapted to animals. Our findings may be utilized in agriculture and bioacoustic procedures.

## 1 Introduction

The domestication of cattle and buffalo is different; while cattle were domesticated approximately 10,000 years ago (1), buffalo were domesticated 3,000–7,000 years ago (2). The water buffalo (*Bubalus bubalis*) is kept for both meat and dairy production (3), with a global population of 202 million individuals worldwide in 2020 (2), making its importance among farm animals indisputable, yet little analysis of its vocalization has been done. A Domesticated cattle and buffalo are highly gregarious and live in herds in nature and on farms (4). In their herds, there is a network between the individuals and they communicate with each other in various ways (5). They can recognize each other visually, by smell or by vocalization. Researched a correlation between the vocalization of cattle and their behavior. They distinguished six behavioral groups: ‘lying & ruminating’, ‘feeding related’, ‘social interaction’, ‘sexual behavior’, ‘stress-related behavior’ and ‘remaining behavior’ (6). Of the different behaviors, vocalization is present in 17.2% (7). The analysis of vocalization is a good opportunity to examine individuals of a herd (8). Cattle vocalization is affected by multiple factors, including farming method (9), age, dominance (10), weight and/or sex (11, 12), estrous climax (13), and castration, which is a highly stressful activity similar to selection (14). Some numerous studies have been published the intense variability between cows (15–17).

Vocalization is triggered by a complex set of hormonal and nervous system reactions. The information on external events that modify emotions, or hormonal and homeostatic factors influencing mood, are perceived by limbic centers of the forebrain. Signals are then transferred via centers of the midbrain (periaqueductal grey) and the lower brainstem to effector muscles of the vocal system (8). Cattle make two main types of calls, forming them by adjusting their supra-laryngeal (above the larynx) vocal sound-producing organ. They use a nasalized low-frequency sound for close contact and an orally produced high-frequency sound for distant communication or for expressing an emotionally aroused state (18–21). Vocalization is one of the most conspicuous behavioral changes in cattle, triggered by a feeling of discomfort (22, 23). As they are prey animals, vocalization within a livestock is minimal (8). When it can be heard, it has an importance from a biological point of view (22), for example in the case of weaning (5, 20, 24). Weaning is stressful for the cows. Oral vocalizations are a sign of stress (25, 26).

In the first hours after calving, vocalization is an important element in the development of the bond between the cow and her newborn calf (27, 28). F0 is the standard symbol for the fundamental frequency. Oral vocalization is triggered by isolation, pain or anxiety (10), while nasal sounds are usually produced in the first hours after calving [F0 = 81.17 ± 0.98 Hz; (12)], often used by dams towards their offspring in combination with licking (29). These nasal sounds also have a calming effect on the cows (30).

The parameters of sounds are affected by the anatomy of the larynx, the length, the thickness and the muscle tension of the vocal folds (31, 32). The filter selectively enhances or dampens specific frequency ranges of the source signal. As a result, a heterogeneous sound spectrum is created containing various formant frequency peaks (32, 33). The sound frequency of dams is higher during weaning too, like at the time of other dam-calf interactions (26, 34). Oral vocalization is generally connected with arousal (31). The frequency of the various sounds of vocalizing cattle is usually between 50 Hz and 1,250 Hz (4). The mean is between 120 and 180 Hz. It can be detected spectrographically up to 7 or 8 kHz; the peak call is in the 350 to 420 Hz range. Sometimes calls below 50 Hz, like the 31 Hz (10) newly weaned calves.

In terms of frequency, a cow’s hearing ranges from 23 Hz to 37,000 Hz (35). The cows have a sound threshold of 85 dB – 90 dB sound pressure level (36), and any range of environmental sounds exceeding 110 dB SPL may cause physical damage (37, 38).

Dams made high-frequency calls (HFC) during weaning, where visual contact was excluded (F0 = 152.8 ± 3.10 Hz) (12). Their normal range in the case of a 60 dB sound is between 23 Hz and 37 kHz. The highest sensitivity is at around 8 kHz (35). This important trait helps them notice predators in time.

The demand for innovative tools that collect and analyze information about the livestock and individuals is increasing. In precision livestock farming (PLF), is increasingly important nowadays, because farmer-cattle interaction is decreasing (39). Research is becoming rather interdisciplinary, aiming to detect connections between vocalization, the nervous system and the hormonal systems. From the point of view of animal welfare, vocalization can be very useful as exact feedback from the animal about its general state. The management of a farm or a plant is decisive for the success of animal welfare (40). The vocal individuality of high-frequency calls of cattle does not change in farming contexts of different emotional valence, including positive and negative emotional valence and situations (19). According to the literature, there is a significant difference between the vocalizations of lying individuals and the sounds recorded during other behaviors (83 ± 4.3 Hz versus 298 ± 8.0 Hz; *p* < 0.05). Mature dairy cattle have a significantly lower maximum frequency (Hz) than heifers (332.6 ± 0.2 Hz versus 218.5 Hz ± 0,3 Hz; p < 0.05) (6). The mean F0 high-frequency vocalization of beef cattle is 153 Hz, the formant frequencies are between 228 and 3,181 Hz, the average duration is 1.2 s and is made orally (12). In PLF the technology constantly measures the variables, such as movements, feed intake or oestrus (41), and thereby helps farmers control their livestock.

The mel frequency cepstral coefficient (MFCC) is regularly used for sound analysis and their categorization or grouping (42); therefore, we also employed it. Such coefficients have acoustic features that can be widely used in various applications later (43–46). The mel scale-based cepstrum can be used well in the recognition of speech sounds. However, the mel scale is a distortion of the frequency scale that is adapted to human hearing; it is a scale with an even pitch sensation (47). The scale is based on people’s binaural hearing. This distortion depends on the shape of the human hearing organ. It is possible to start from the assumption that the hearing organ of these big animals differs only in size from that of humans. Mel frequency cepstrum analysis of the sounds indicated oestrus with 94% accuracy (44) other authors have achieved even better accuracy, 97% (48). Automated software is able to detect behavioral changes early, which in turn can lead to early responses, even to the treatment of a disease, thus reducing labor time and veterinary costs (49). Accurate knowledge of vocalization can be such a measure. Vocalization is an excellent tool for detecting animal welfare problems, in the field of agriculture; especially where individuals are not always visible, it can be used to assess animal welfare. The collection, observation and assessment of vocalization do not require animal interaction and are therefore stress-free. However, to do this, bovine vocalization software needs as accurate data as possible (50). Although analyzing vocalization is useful, limited research has been done on the analysis of the vocalization of cattle and its categorization. Further research is needed on vocalization analysis in order to reduce stress on animals and its temporal effects (10, 51). This paper analyses the sounds of grey cattle and water buffalo, among others, using the mel scale cepstrum. The cows that we studied vocalized in the weaning stress situation (52). We also aimed to detect differences in vocalization between the two species and between individuals (oral vocalization).

## 2 Materials and methods

### 2.1 The process of data collection

Our research site is a farm called Szamárhát, owned by Tiszatáj Közalapítvány, a public foundation. There, animal farming is extensive from spring to autumn and in winter, at the time of recording the sounds, the individuals are in barns. In 2019 and 2020 in the wintertime we recorded 14 calls of water buffalo cows and 13 calls of grey cattle cows. We applied multiple methods for the sound analysis of the 139 sound pieces. In MATLAB R20201a, we used the Polyfit function (sixth degree polynomial), in addition to spectrum analysis, mel-scale cepstrum and formant analysis. As prey animals, they vocalize only when justified (8), so cattle and buffalo cows had to be encouraged to vocalize. The situation was the same during each data collection. The audio recording took place between 8:00 and 11:00 am. Within 2–12 h after calving, the calves were taken for weight measurement and ear tagging. At that moment, a gate was placed between the dam and the offspring, as a physical barrier. The individuals heard and saw each other but were unable to physically contact each other. The dams made oral vocalization, a sign of stress (52). The vocalizing dams were standing. The eldest cow was 13 years old, the youngest one was 5. Each individual had already calved multiple times in the past. The sounds were recorded with a Sony IC Recorder AX412F whose sample rate is 32.000 Hz. Every time, we directed the recorder toward the cow from a distance of 2–3 meters. We aimed to avoid any interfering noise.

### 2.2 Statistical analysis

**Figure 1:**
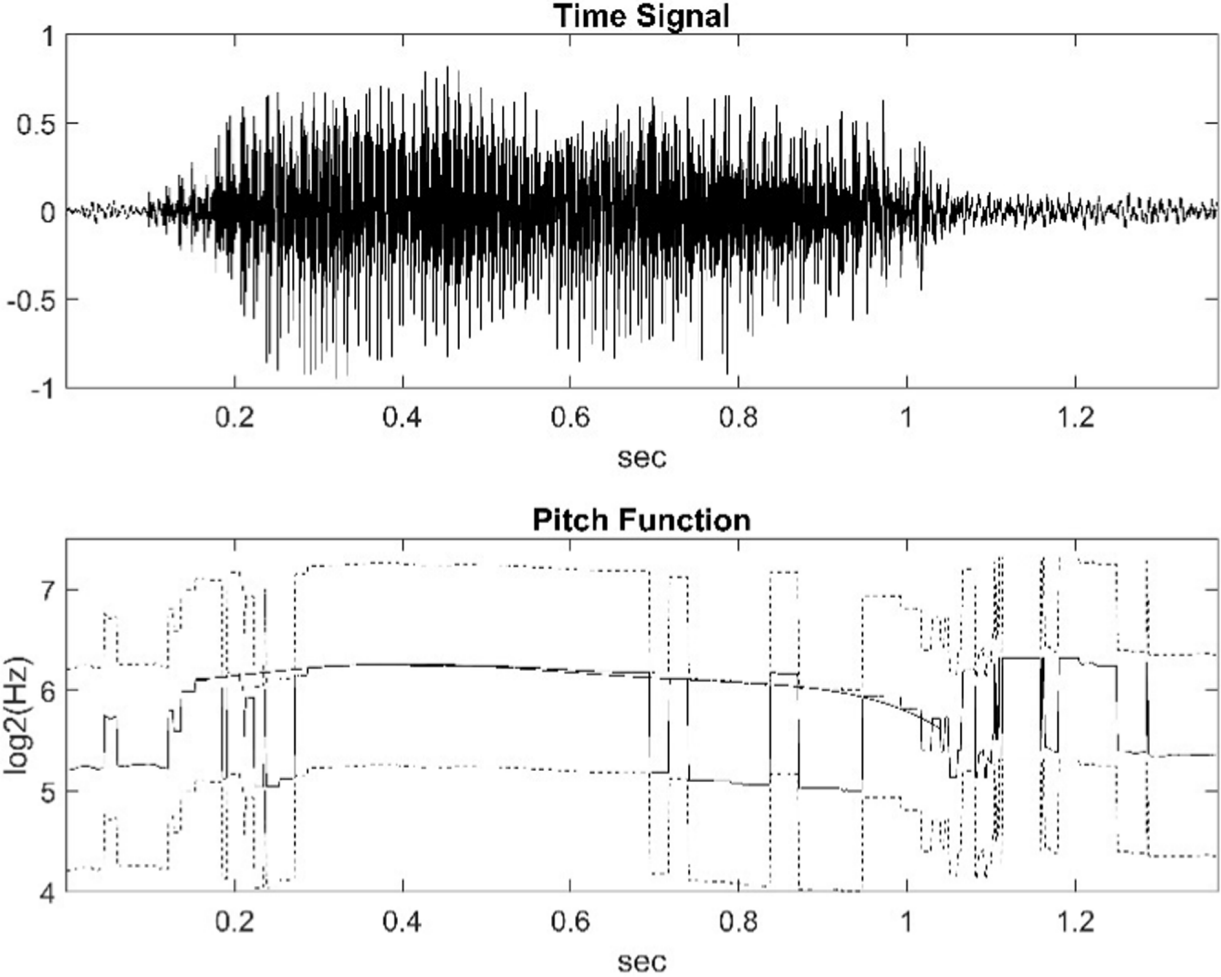
The upper part of the figure shows the waveform of a water buffalo call. The lower part is its pitch function (solid line). Due to the usual octave jump, it is shifted up and down by one octave (dashed line). The function of the values we deemed correct was smoothed by fitting a suitable 6th degree polynomial.

To determine the fundamental period or frequency of quasi-periodic signals, we utilized the Pitch function from the Matlab Audio Toolbox, which offers five methods. This function offers five methods. Among them, the Normalized Correlation Function (NCF) appeared to be the most appropriate (53), although it was often inaccurate (octave jump, see, Figure 1). We applied the Mann–Whitney U test to the samples. We examined the pitch using a two-sample t-test with the logarithm of the value.

## 3 Results and discussion

Cattle vocalizations provide essential information about the individuals. If we learn how to interpret this information correctly, it can be used to improve the management or welfare assessment. When under stress, cattle make sounds with a specific pattern (54). Despite the numerous studies published about the acoustic characteristics of such sounds (4, 10), there has not been an acoustic analysis regarding the behavior and the condition of cattle in isolation (55). Such an analysis could serve as a useful data source for surveillance systems (6). The bellow and the vocalization of grey cattle and water buffalo are similar to the vowels of human speech: they can be considered quasi-periodic signals, i.e., sound signals that cause a sense of pitch. While the vowels in speech are relatively short, the sounds of animals are long-lasting and quasi-stationary, i.e., their spectral properties change slowly and little. The typical number of shapes in a formant analysis of speech sounds is 6. Of these, only the first three characterize the speech sound, the others characterize the speaker. On this basis, five formants were considered sufficient for comparing the voices of the two species. We performed a mel scale-based cepstrum analysis of buffaloes and grey beef cows.

### 3.1 Intonation of vocalizations

Figure 1 shows the waveform of the vocalization of a water buffalo and the graph icon (solid line) of the pitch function. On the y-axis, the unit is the logarithm of the octave as a frequency ratio. In addition to the value provided by the pitch function, the figure contains the graph shifted up and down by one octave (dashed line). In our study, the fundamental frequency was partly automatically determined as a function of time, and then we fitted a sixth-degree polynomial to its logarithm (thick line). The result produced with NFC is the thin, continuous line the jumps one octave. This can be corrected with MATLAB.

**Figure 2:**
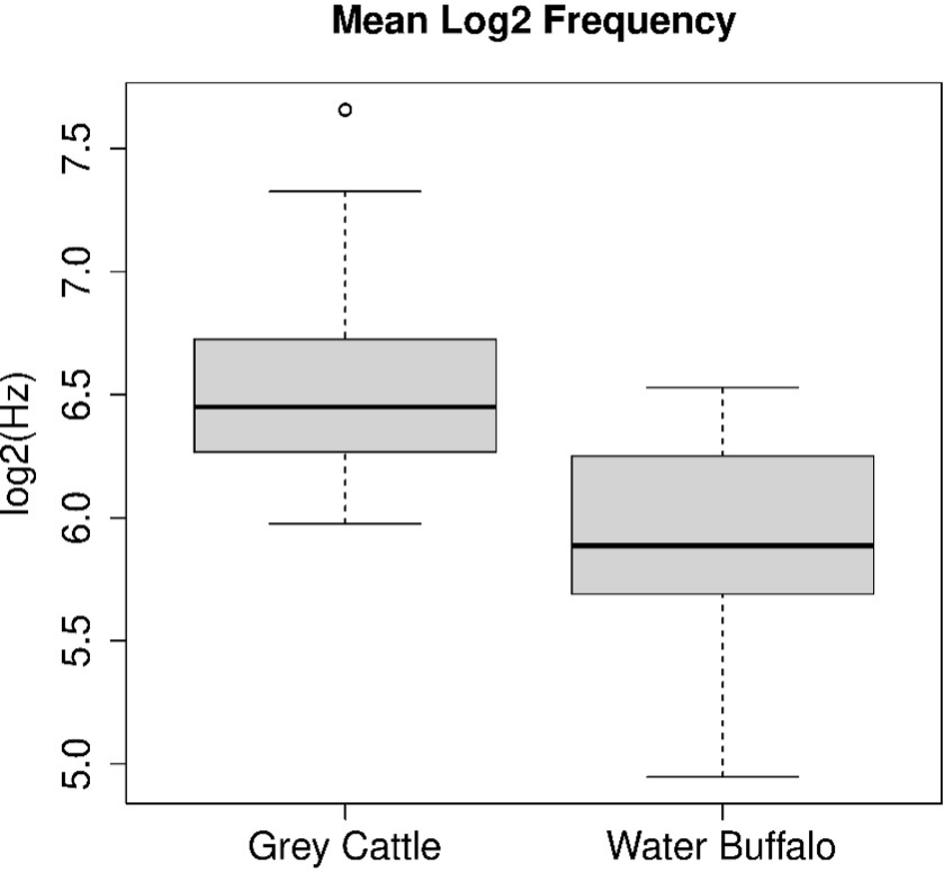
The mean pitch of the vocalizations was examined separately for the two animal species. The Mann–Whitney U test, which we applied to the two samples, refuted (*p*-value = 7.52e−14) that the two samples come from the same distribution and that the two means are the same. Figure shows that the pitch of grey cattle’s vocalizations is significantly higher than that of water buffalos.

The pitch of the vocalizations was examined separately for the two animal species. Some animal vocalizations do not have a constant pitch, they change slowly over time. The pitch values were determined the log2(Hz) scale. The mean pitches of each vocalization form the two samples for the two animal species. The Mann–Whitney U test, which we applied to the two samples, refuted (*p*-value = 7.52e−14) that the two samples come from the same distribution and that the two means are the same. Consequently, there is a significant difference between the pitches of the vocalizations of the two species. The two medians are 6.579 log2 (Hz) and 5.887 log2 (Hz) (i.e., 95.6 Hz and 59.18 Hz), their difference is 0.692 octaves, i.e., 4.15 whole intervals. The temporal means of the grey cattle’s vocalizations are on average much higher (Figure 2).

**Figure 3:**
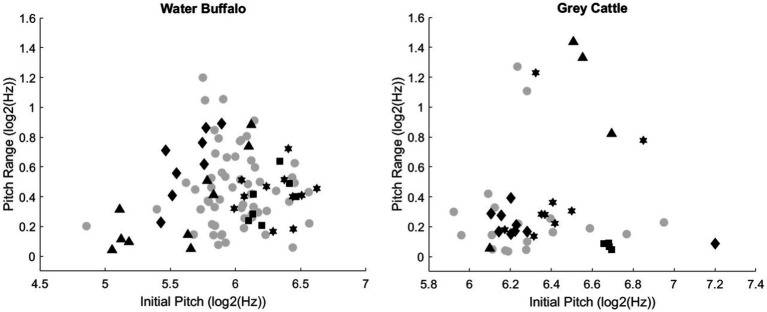
The relationship between the initial pitch and the sound range of the vocalizations. The grey circular points indicate the distribution of the value pairs (initial pitch, sound range) in general. The various black marks also belong to one vocalization, and the same marks are different vocalizations of the same animal. The figures show that there is a large standard deviation for the individuals.

The study also analyzed the pitch of the vocalizations and their range (Figure 3). The grey, circular points are the value pairs of a smaller population, they are meant to indicate the distribution of the value pairs in general. The different black marks also belong to one vocalization, and the same marks are different vocalizations of the same animal. The figures show that there is a large standard deviation for the individuals. Based on this methodology, we did not find differences between individuals of one species.

**Figure 4:**
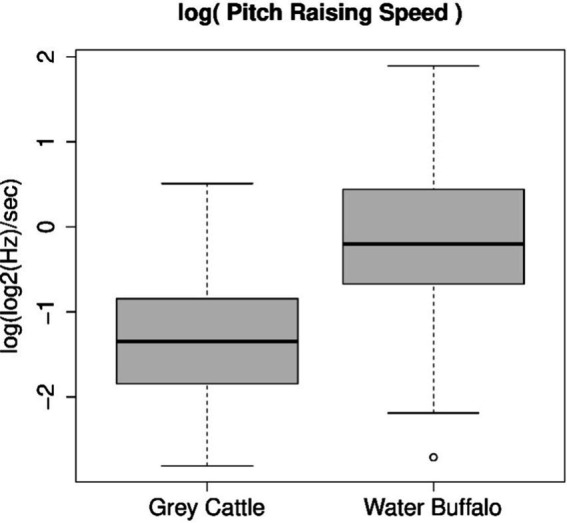
The logarithm of the average rate of pitch change follows a normal distribution for both animal species. The Shapiro–Wilk normality test, which we applied to both samples, did not refute this in either case; therefore, we could apply the Welch Two Sample *t*-test and found that the dynamics of vocalizations are significantly different for the two animal species. The figure shows the result of this statistical examination. The tune of buffalo vocalizations is three times more dynamic than that of grey cattle.

We examined the average rate of pitch change. The pitch was again taken into account in log2(Hz). In the case of grey cattle and buffaloes, instead of the samples consisting of average rate values, the samples consisting of their logarithms can be considered as normally distributed. The applied Shapiro–Wilk normality test did not refute this in either case (*p*-value = 0.6494 or *p*-value = 0.8553). Applying the Welch Two Sample t-test to the two samples, we discarded the assumption that the medians of the two samples are the same (*p*-value = 2.36e-10). Figure 4 shows the parameters of the samples. The two medians are-1.349 log(log2(Hz)/s) and-0.199 log(log2(Hz)/s). Accordingly, the geometric means of the average rates of changes (we can now switch from log(log2(Hz)) to octaves) are 0.259 octaves/sec and 0.820 octaves/sec, which means that the average pitch change in 1 s is 1.6 whole intervals for grey cattle and 4.9 whole intervals for buffaloes. The tune of buffalo vocalizations is three times more dynamic than that of grey cattle.

**Figure 5:**
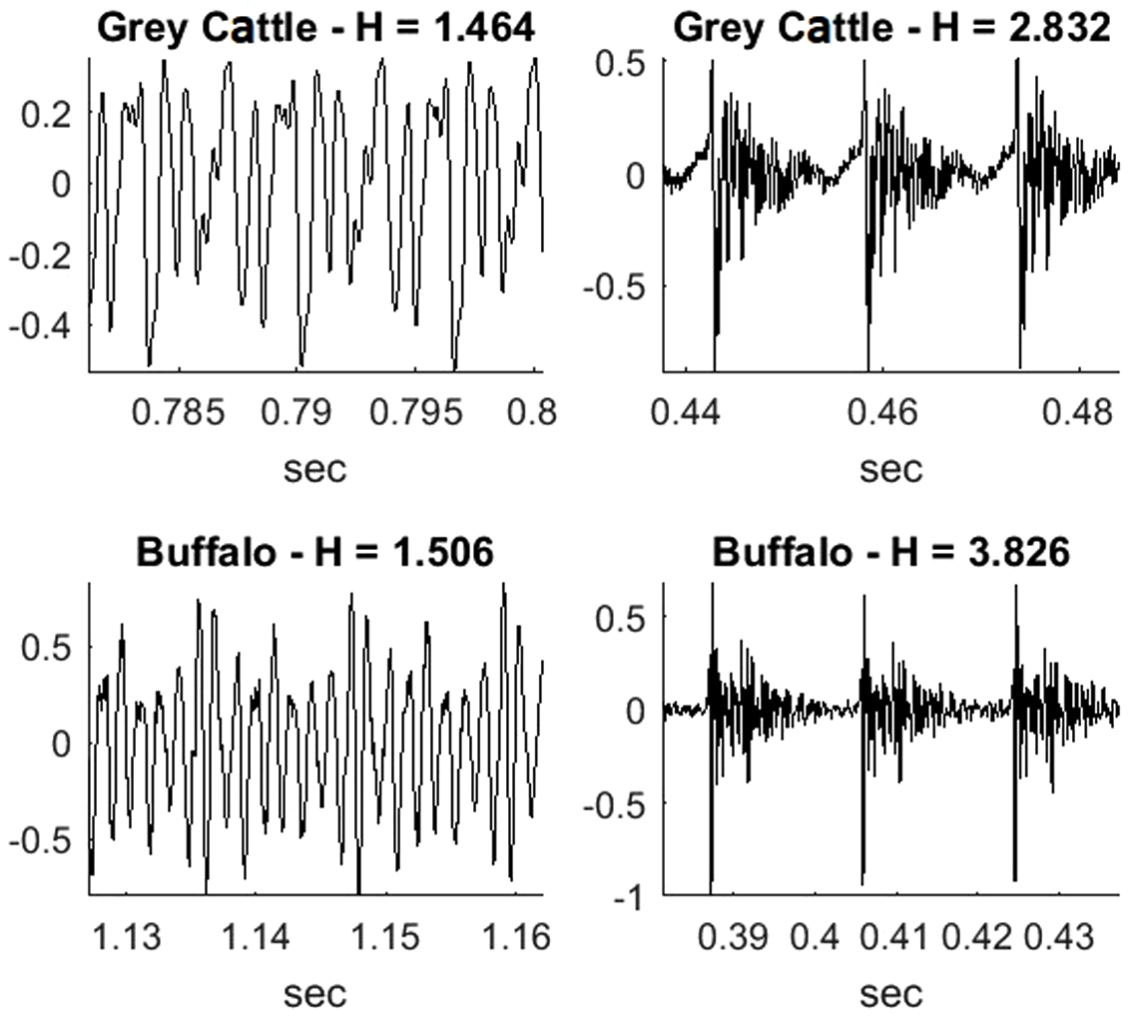
The degree of unevenness of the energy within the fundamental period was defined as the quotient of the 4-and 1-parameter power means. It shows three periods of vocalization belonging to the smallest and the greatest value, H = energy unevenness.

The pitches are relatively low; consequently, it is particularly interesting when the energy is high at a few points in a period only. At such points, the sound resembles the rattle of a gun, although the lower limit of the hearing range is still ‘far away’ on the frequency scale. The extent to which the instantaneous energy is only high at a few points in one period of the waveform can be expressed by using power means. The property of power that we used is that the greater the parameter of the mean, the greater the weight with which greater values are included in the mean value. This property of power means is also used in the echo cancellation (Foundation, J.-M. V., www.speex.org) module of the speex codec program. The argument of the power mean will be the point-by-point square of one period of the quasi-periodic signal. We assumed that these values are all positive, so their power means can be formed. The degree of unevenness of the energy within the fundamental period was defined as the quotient of the 4-and 1-parameter power means. Figure 5 shows three periods of vocalization belonging to the smallest and the greatest value, H = energy unevenness.

We examined the relationship between these values for the two species. The energy unevenness measures of the vocalizations form the two patterns for the two animal species. The Mann–Whitney U test refuted (p-value4 = 0.001356) that the two samples come from the same distribution and that the two averages are the same. The averages are H_grey cattle_ = 1.895, H_water buffalo_ = 2.176.

The quality of animal vocalizations can be examined from the point of view of how much they move away from the continuous sound and become similar to a gun rattle, and how uneven the energy becomes within the fundamental period. To measure this, we introduced the quotient of two power means. The parameters of the power means are 4 and 1, and they refer to the energy values within the fundamental period. The figure shows three fundamental periods of four vocalization waveforms. The relevant energy unevenness quotient (H) is written above the graphs. As the figure shows, the more uneven the energy is, the higher is this value, for both grey cattle and water buffalo.

### 3.2 Spectral-based methods

**Figure 6:**
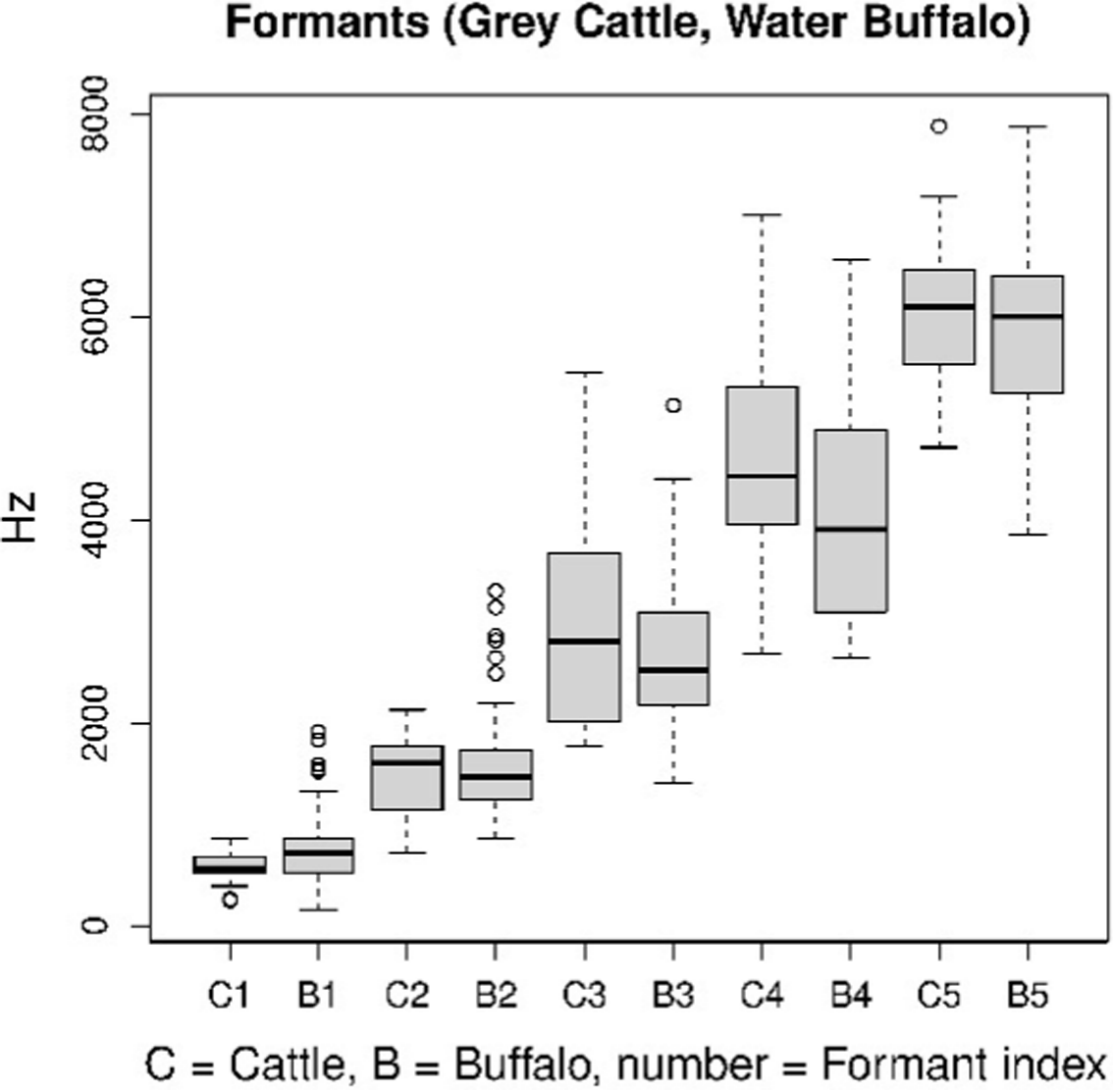
The formant structures of vocalizations as quasi-periodic signals are standard characterizations of such signals. The figure shows that no significant difference between the two animal species can be detected using this method.

Vowels are characterized by their formant structure. Only certain sounds in human speech are quasi-periodic. Vowels and voiced consonants are flexible. Sound recognition based on formant structure only works in the case of vowels. This is what we applied to animal vocalizations, to their quasi-periodic details. In the study, five formant frequencies were determined separately for grey cattle and water buffaloes. The formant structure is compressed by the spectrum into a number, into a series of 5–6 elements of formant frequency and width value pairs. Frequency is the more significant component of the pairs. As the function of frequency, the spectrum peaks at points that correspond to the formant frequencies. One of the characteristics of hearing is that the peaks overshadow their surroundings. This is the reason why we deal with the peaks only, concerning the sense. The width can determine the height of the peaks. In formant structures, the pairs are in an increasing order based on frequency. Figure 6 shows the parameters of the distributions (C1, B1), the pairs denote the median of the formant frequencies, separately for beef and buffalo. Only those columns can be compared that have the same index and do not differ significantly (Figure 6). Perhaps this method could be used to identify individuals, but there are not enough samples from each individual in this study.

### 3.3 Mel scale cepstrum

Some studies have already incorporated human-derived algorithms to analyze and then recognize cattle vocalizations (44, 56). The MATLAB mfcc function returns the mel frequency cepstral coefficients (MFCCs) for the audio input (57).

**Figure 7:**
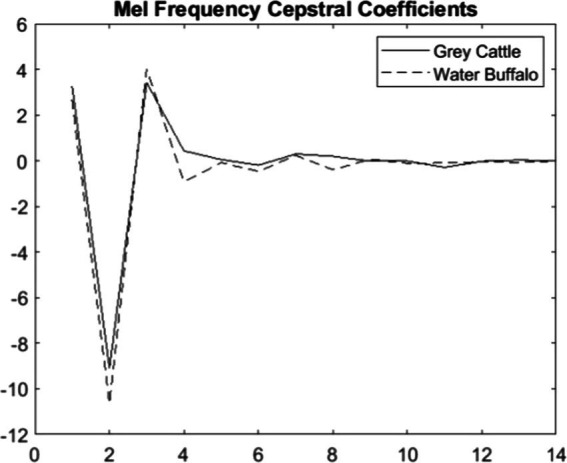
The averages of the mel frequency cepstral coefficients of the vocalizations show significant differences in the case of two coefficients, the 4th and 8th coefficients.

The fundamental parameters of the function are the sound sample and the sampling frequency. Instead of the Fs actual sampling frequency, we chose Fs’ = 0.62*Fs so that the values of the members of the two populations are separated as much as possible. This value corresponds to what is known about the hearing range of animals, that the upper limit is around 30–40 kHz. The averages of the coefficients are 14–14 for cattle and buffalos, respectively. However, these averages are roughly the same for the two populations, except for the fourth and the eighth (Figure 7).

**Figure 8:**
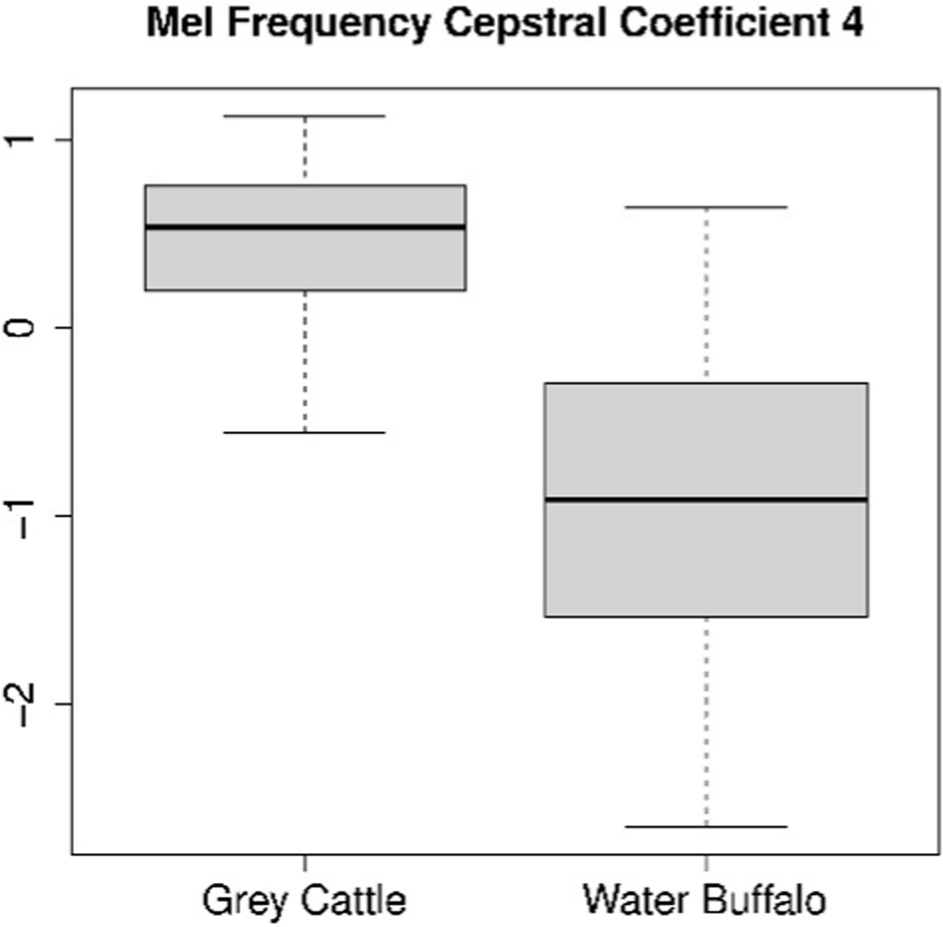
The result of the statistical tests for the 4th mel frequency cepstral coefficient of vocalizations: in the case of grey cattle, this coefficient is significantly greater than that of water buffalo.

**Figure 9:**
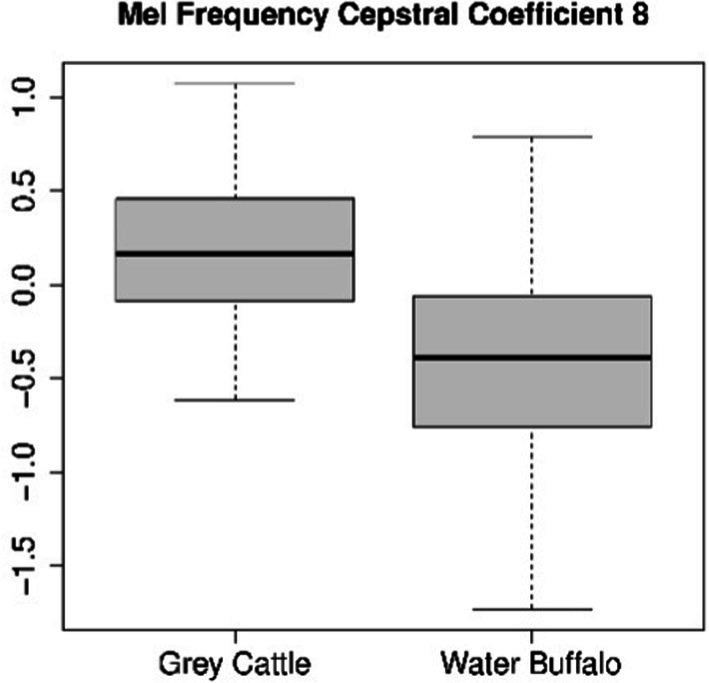
The result of the statistical tests for the 8th mel frequency cepstral coefficient of vocalizations: in the case of grey cattle, this coefficient is significantly greater than that of water buffalo.

**Figure 10:**
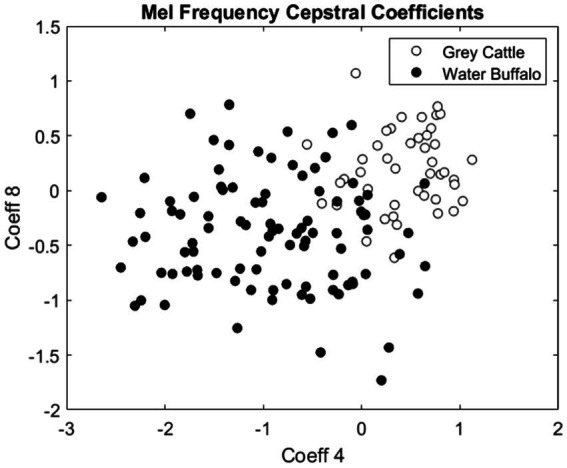
The pairs consisting of the 4th and 8th coefficients of the mel frequency cepstral coefficients of the vocalizations. The empty circles represent the vocalizations of grey cattle and the filled circles are related to water buffalo.

The difference can be verified statistically too. For both coefficients 4 (Figure 8) and 8 (Figure 9), the Mann–Whitney U test refuted (*p*-value_4_ = 2.2e-16, p-value_8_ = 5.9e-10) that the two samples come from the same distribution and that the two means are the same. The medians are M4_grey cattle_ = 0.536, M4_water buffalo_ = −0.910, M8_grey cattle_ = 0.168, M8_water buffalo_ = −0.389. We also demonstrate the distribution of coefficient pairs. (Figure 10).

## 4 Conclusion

The findings of bioacoustic studies of cattle have mainly been published concerning dairy cattle, focusing on the sounds that cattle make in response to weaning. To make use of such findings in advanced milk producing procedures in the future, it is necessary to better understand the sound kit of cattle and to interpret the information that the sounds convey. The specific acoustic parameters of the various cattle breeds need further research so that the species or breed effects can be excluded. In a large number of cattle farm animals neurobiological research is a difficult task that probably can be performed only with a small number of individuals in specialized laboratories. In this paper, we have analyzed the stress-induced oral vocalizations of water buffaloes and grey cattle using different methods. Concerning the acoustic features of vocalization, our data do not confirm the intense variability between cows, about which numerous studies have been published, but the difference between the two species was consistently detected. The pitch of individuals of the two species that we studied varies over time. The perception of pitch in terms of logHz value becomes a smooth variable, thus showing that the buffalo voice is three times more dynamic than that of the grey cattle. Our results can be utilized in the field of agriculture, for bioacoustic procedures.

## Data Availability

The original contributions presented in the study are included in the article/supplementary material, further inquiries can be directed to the corresponding authors.
